# Selenite Substituted Calcium Phosphates: Preparation, Characterization, and Cytotoxic Activity

**DOI:** 10.3390/ma14123436

**Published:** 2021-06-21

**Authors:** Antonia Ressler, Maja Antunović, Matija Cvetnić, Marica Ivanković, Hrvoje Ivanković

**Affiliations:** Faculty of Chemical Engineering and Technology, University of Zagreb, Marulićev trg 19, p.p.177, HR-10001 Zagreb, Croatia; maja.antunovic2007@gmail.com (M.A.); mcvetnic@fkit.hr (M.C.); mivank@fkit.hr (M.I.); hivan@fkit.hr (H.I.)

**Keywords:** biomaterials, hydroxyapatite, ionic substitutions, octacalcium phosphate, osteosarcoma, selenite

## Abstract

The aim of this study was to prepare a biomimetic selenium substituted calcium phosphate system for potential application in osteosarcoma therapy. Calcium phosphate (CaP) systems substituted with selenite ions were prepared by the wet precipitation method, using biogenic CaCO_3_ (derived from cuttlefish bone), CO(NH_2_)_2_-H_3_PO_4_, and Na_2_SeO_3_·5H_2_O as reagents. Starting reaction mixtures were prepared based on the formula for selenite-substituted hydroxyapatite, Ca_10_(PO_4_)_6-*x*_(SeO_3_)*_x_*(OH)_2_, with Ca/(P + Se) molar ratio of 1.67 and Se/(P + Se) molar ratio of: 0, 0.01, 0.05, and 0.10, respectively. The prepared CaP powders were characterized by Fourier transform infrared spectrometry, elemental analysis, scanning electron microscopy, X-ray powder diffraction analysis and Rietveld refinement studies. Phase transformation and ion release were analyzed during 7 days of incubation in simulated body fluid at 37 °C. The metabolic activity of healthy and osteosarcoma cell lines was assessed by cell cytotoxicity and viability test. The as-prepared powders were composed of calcium-deficient carbonated hydroxyapatite (HAp), octacalcium phosphate (OCP), and amorphous calcium phosphate (ACP). Along with the selenite substitution, the presence of Sr^2+^, Na^+^, and Mg^2+^ was detected as a result of using cuttlefish bone as a precursor for Ca^2+^ ions. Inductively coupled plasma mass spectrometry analysis showed that the Se/(P + Se) molar ratios of selenite substituted powders are lower than the nominal ratios. Heat treated powders were composed of HAp, *α*-tricalcium phosphate (*α*-TCP) and *β*-tricalcium phosphate (*β*-TCP). Doping CaP structure with selenite ions improves the thermal stability of HAp. The powder with the Se/(P + Se) molar ratio of 0.007 showed selective toxicity to cancer cells.

## 1. Introduction

Until recently, hydroxyapatite (HAp, Ca_10_(PO_4_)_6_(OH)_2_) was considered to be the main inorganic component of bone tissue. However, recent studies and advanced techniques have shown that biological apatite is characterized by a more complex structure [1]. Firstly, amorphous calcium phosphate (ACP) and octacalcium phosphate (OCP) are formed and apatite is precipitated through phase transformation at physiological conditions [2]. Biological apatite is non-stoichiometric carbonated hydroxyapatite with a considerably reduced content of calcium and hydroxyl groups. Its structure is multi-substituted with various ions, primarily magnesium (Mg^2+^), sodium (Na^+^), strontium (Sr^2+^), potassium (K^+^), zinc (Zn^2+^), manganese (Mn^2+^), carbonate (CO_3_^2−^), and hydrogen phosphate (HPO_4_^2−^). The trace elements substituted in HAp lattice affect its properties such as the crystal size, crystallinity level, agglomeration tendency, solubility, morphology, lattice parameters, thermal properties, and bioactivity [3,4].

Bone cancer is one of the most prevalent cancers with the lowest long-term survival rate. Osteosarcoma is an immensely malignant bone tumor that happens most often in young children, adolescents, and young adults. The current treatment of osteosarcoma includes surgery, radiotherapy, and chemotherapy [5]. The HAp itself has strong apoptosis induction efficacy on many cancer cells [6]. However, to create more functionalized calcium phosphate (CaP) materials, modification studies have attracted attention, especially doping the structure with target ions [7].

Selenium is an essential element for the proper functioning of bone tissue with strong antioxidant properties [1,6]. There have been reports on the positive effects of selenium on cardiovascular diseases, cancers, the brain, reproduction, viral infections, and bone tissue [1]. Selenium may play an important role in protection against osteoarthritis, rheumatoid arthritis, or osteoporosis [1]. Selenium deficiencies may delay growth and affect the metabolism of bone tissue [1]. The mechanism of these processes is associated with the function of selenoproteins, of which at least nine are expressed in human fetal osteoblasts. Their expression seems to protect bones from oxidative stress, which is important in the regulation of inflammation and the differentiation of bone cells [1,5]. In recent years, selenium has attracted attention as CaP substituent as it can induce tumor cell apoptosis [7]. In vitro and in vivo studies obtained by Wang et al. confirmed that Se-substituted HAp has antitumor activity and can induce the apoptosis of human osteosarcoma cells [8]. HAp substituted with selenite ions had pronounced inhibitory effects on the osteosarcoma cell line, while leaving regular fibroblasts intact as reported by Uskoković et al. [9]. However, the cytotoxicity of SeO_3_^2−^-substituted HAp relies on its pH-dependent release into the medium and a high concentration of selenium is suggested to induce non-selective cytotoxicity [9]. As reported, a high SeO_3_^2−^-content (3.0 wt%) induced non-cancerous MC3T3-E1 cell death with abnormal morphological changes as early as 24 h of treatment and decreased the expression of bone γ-carboxyglutamate protein 3 (BGLAP3) [9].

Selenium doped HAp is most frequently obtained by wet precipitation methods, using SeO_3_^2^^−^ ions as a source of selenium [6,7,8,9,10]. To the best of our knowledge no study on synthesis and characterization of Se-substituted CaPs, derived from cuttlefish bone, has been reported. The use of natural resources such as cuttlefish bone for preparing CaPs is a promising concept since these resources bring, besides the calcium, many beneficial ions found in human bones. In the present research, Se-substituted CaPs powders (CaP_Se) were synthesized by precipitation method at mild conditions. The effect of Se-substitution on quantitative phase content, lattice parameters, thermal stability, and cell viability of healthy and osteosarcoma cells have been studied. Additionally, an ion release study and a phase transformation after 7 days of incubation in simulated body fluid at 37 °C were conducted.

## 2. Materials and Methods

### 2.1. Synthesis of Selenium Substituted Calcium Phosphates

As a source of Ca^2+^ ions, CaCO_3_ was used, obtained from cuttlefish bone as described in our previous paper [11]. The non-substituted CaP_0Se system was prepared by adding urea phosphate (UPH, CO(NH_2_)_2_-H_3_PO_4_, Sigma Aldrich, St. Louis, MO, USA) into dissolved/suspended CaCO_3_ at 50 °C, while stirring was continued for 4 days followed by overnight aging at room temperature (T = 23.0 ± 0.2 °C). In order to obtain a Se-substituted CaP system, sodium selenite pentahydrate (Na_2_SeO_3_·5H_2_O, Sigma Aldrich, St. Louis, MO, USA) was added before CaCO_3_ at 30 °C. Four samples with different Se/(P + Se) molar ratios (0, 0.01, 0.05, and 0.10) have been prepared and labelled CaP_Se0, CaP_Se1, CaP_Se5, and CaP_Se10, respectively. According to the literature [10], in the selected range of Se/(P + Se) molar ratios, no secondary phases (other than hydroxyapatite) were expected. The Ca/(P + Se) molar ratio was fixed at 1.67. The corresponding amount of the reagents in starting reaction mixtures are listed in Table 1. Supernatant and CaP particles were separated by filtering without washing. Parts of the as-prepared powders were sintered at 1200 °C for 2 h. Finally, part of obtained as-prepared CaP, CaP_Se0 and CaP_Se5 powders were incubated in simulated body fluid (SBF, reagents were purchased from Sigma Aldrich, St. Louis, MO, USA) for 7 days at 37 °C. Non-substituted CaP powder obtained from synthetic CaCO_3_ as described in [11] was used as control.

### 2.2. Characterization of Selenium Substituted Calcium Phosphates

The final pH of prepared CaP suspensions was measured on a Schott CG 842 pH-meter (Schott, Mainz, Germany) using a BlueLine 14 electrode with a precision of 0.01 at room temperature (T = 23.0 ± 0.2 °C).

The elemental content of prepared CaP enriched with selenium oxyanion was analyzed by inductively coupled plasma mass spectrometry (ICP-MS), using a PerkinElmer SCIEXT ELANR DRC-e (PerkinElmer, Concord, ON, Canada). The analyses were performed according to the manufacturer’s protocol as described in our previous paper [11].

The Fourier transform infrared spectra (FTIR) was carried out using an attenuated total reflectance (ATR) spectrometer for solids with diamond crystal (Bruker Vertex 70, Bruker optics, Ettlingen, Germany) at 20 °C over the spectral range of 4000–400 cm^−1^, with 32 scans and 4 cm^−1^ of resolution.

The powder morphology was analyzed by scanning electron microscopy (SEM, TESCAN Vega3 EasyProbe, Kohoutovice, Czech Republic) at an electron beam energy of 13 keV. Dried powders were coated with plasma of gold and palladium for 60 s.

The as-prepared and heat-treated samples mixed with 5 wt% of polycrystalline silicon (NIST SRN 640e, Sigma Aldrich, St. Louis, MO, USA) were characterized by X-ray diffraction (XRD) analysis using a Shimadzu XRD-6000 diffractometer (Shimadzu, Duisburg, Germany) with Cu K_α_ (1.5406 Å) radiation operated at 40 kV and 30 mA. The diffraction patterns were collected in the 2*θ* range 3–60° for as-prepared and 20–70° for heat treated samples, with a step size of 0.02° and exposure of 10s.

### 2.3. Rietveld Refinement Studies

The obtained collection of X-ray powder diffraction pattern data was used for Rietveld refinement studies performed by means of the software DIFFRAC.SUITE TOPAS V.5.0. (Bruker, Karlsruhe, Germany) with the fundamental parameters approach. Additionally, the Si-standard was used to estimate the instrumental parameters (e.g., 2*θ* correction). The structural parameters of HAp, reported by Veselinović et al. [12], OCP by Espanol et al. [13], *β*-tricalcium phosphate (*β*-TCP) by Yashima et al. [14], and *α*-tricalcium phosphate (*α*-TCP) by Mathew et al. [15] have been used as the initial values in the refinements. Refined parameters were phase weight percentage, lattice parameters, scale factor, and specimen displacement. The weighted profile R-factor (R_wp_) and expected R factor (R_exp_) were used to assess the goodness-of-fit of the Rietveld refinement. The results with R_wp_ < 10% and R_exp_ < 3% were considered to be acceptable.

### 2.4. Ion Release Study

To examine in vitro ion release, as-prepared CaP, CaP_Se0 and CaP_Se5 powders were incubated in static simulated body fluid (SBF, pH 7.4) at 37 °C. The SBF solution was prepared as previously described by Bohner and Lemaitre [16]. 100 mg of powders were immersed in 10 mL of SBF for 7 days. At determined time points, the concentration of Se ions was determined by ICP-MS analysis, using a PerkinElmer SCIEXT ELANR DRC-e. The pH value of SBF during incubation was measured on a Schott CG 842 pH-meter using a BlueLine 14 electrode with a precision of 0.01 (T = 37.0 ± 0.5 °C). After incubation, samples were washed in demineralized water and dried at room temperature (T = 23.0 ± 0.2 °C). Dried samples were mixed with 5 wt% of polycrystalline silicon (NIST SRN 640e, Sigma Aldrich, St. Louis, MO, USA) and an XRD analysis was performed using a Shimadzu XRD-6000 diffractometer in the 2*θ* range 3–60°, with a step size of 0.02° and exposure of 10 s.

### 2.5. Anticancer Studies

#### 2.5.1. Preparation of Extracts of CaP Powders and Cell Culture Conditions

To determine selective anticancer properties and biocompatibility of as-prepared powders (CaP_Se0, CaP_Se1, CaP_Se5, CaP_Se10) human embryonic kidney 293 (HEK 293) and human osteosarcoma (U2OS) cells were used. Cells were kindly provided by prof. Inga Urlić, Faculty of Science, University of Zagreb. Non-substituted CaP powder obtained from synthetic CaCO_3_ as described in [11] was used as control. Calcium phosphate powders were sterilized for 15 min under UV light and incubated with Dulbecco’s modified Eagle’s culture medium (DMEM)—high glucose (Sigma-Aldrich, St. Louis, MO, USA), supplemented with 10% fetal bovine serum (Capricorn Scientific, Ebsdorfergrund, Germany) and 1% penicillin/streptomycin (Lonza, Basel, Switzerland) at a concentration of 10 mg/mL and kept at 4 °C for 24 h. After incubation, powders were centrifuged (300× *g*) for 5 min, and the supernatant was used for biocompatibility testing.

The HEK 293 and U2OS cell lines were kept in cell culture conditions (5% CO_2_ humidified atmosphere at 37 °C) and fed with culture medium until confluence. When they reached 80% confluence, cells were trypsinized with Trypsin/EDTA (Sigma-Aldrich, St. Louis, MO, USA) to obtain cell suspension. The cells were seeded into each well of a 96-well plate (Corning – Sigma Aldrich, St. Louis, MO, USA) at a concentration of 0.5 × 10^5^ cells per 200 μL of the medium and incubated in a 5% CO_2_ humidified atmosphere at 37 °C for 24 h. After 24 h cell culture, the medium was removed followed by addition of 200 µL of supernatant from calcium phosphate powders to each well.

#### 2.5.2. Cell Viability Evaluation by MTT Assay

Viability of HEK 293 and U2OS cells was obtained using MTT (3-(4,5-dimethylthiazol-2-yl)-2,5-diphenyltetrazolium bromide) assay (Sigma-Aldrich, St. Louis, MO, USA), after 1 and 3 days of cell treatment with the extract of as-prepared powders. After determined time points, the medium was removed and 40 μL of MTT solution was added to each well. After 3 h at 37 °C, 170 μL of dimethyl sulfoxide (DMSO, Sigma-Aldrich, St. Louis, MO, USA) was added to each well. When formazan crystals were dissolved, the solution was set for colorimetric detection at 560 nm using a microplate reader (GlowMax-Multi, Promega, Madison, WI, USA). All experiments were performed in triplicates. The percentage of cell viability was calculated from the absorbance readings in reference to untreated cells.

### 2.6. Statistical Analysis

All biological experiments were conducted in triplicate. Quantitative results are expressed as mean ± standard error of the mean (*n* = 3). Statistical analysis was performed using one-way ANOVA test with value *p* < 0.05 and *p* < 0.01 considered statistically significant compared to untreated cells (control).

## 3. Results and Discussion

Selenium-substituted calcium phosphate powders (CaP_Se) were synthesized by wet precipitation method at mild conditions and subjected to chemical, crystallographic and morphological characterization.

### 3.1. The Chemical Composition of as-Prepared Powders

The chemical composition of as-prepared powders was determined by ICP-MS analysis. As shown in Table 2 in addition to Ca, P and Se the presence of strontium (Sr^2+^), magnesium (Mg^2+^) and sodium (Na^+^) ions has been detected. Using a biogenic source (cuttlefish bone) as a precursor of Ca^2+^ ions results in a multi-substituted CaP system as already reported in our previous research [11]. Sr^2+^ and Mg^2+^ substitution level is ~0.47 and ~0.45 mol%, respectively, while the substitution level of Na^+^ ion increase from 1.45 mol% (CaP_Se0) to final 3.49 mol% (CaP_Se10). Increase of Na-substitution along with the increase of SeO_3_^2−^ ion content could be a result of charge defect compensation, since the substitution of a double-charged selenite ion for a triple-charged phosphate ion creates a negatively charged vacancy. The Sr^2+^, Mg^2+^ and Na^+^ are typical substitutional ions for Ca^2+^ in biological apatites. The Sr^2+^ ion stimulates the formation of the osteoblast cell line while having an inhibitory effect on osteoclast cells. The Mg^2+^ ion is an essential element, highly important in the early stage of bone formation due to grow factor effect. Prolonged Sr^2+^ and Mg^2+^ deficiency directly result in a decrease in bone density and can cause osteoporosis [17].

As shown in Table 2, the Se/(P + Se) molar ratios in as-prepared powders increase along with the selenium content added to the reaction mixture. However, the Se/(P + Se) molar ratios are lower than the nominal ratios indicating that part of SeO_3_^2^^−^ ions remained in the mother liquor solution after precipitation. The data from Table 2 show that 63.6% of nominally added SeO_3_^2^^−^ ions were incorporated in CaP_Se1, 59.2% in CaP_Se5, and 60.4% in CaP_Se10. Incomplete incorporation of SeO_3_^2^^−^ ions into precipitated calcium phosphates are in accordance with results obtained by Uskoković et al. [9], Wei et al. [10], and Liu et al. [18]. Due to minor substituents, the Ca/(P + Se) molar ratio in starting reaction mixture is lower (~1.61) than the 1.67 required for stoichiometric HAp. In spite of this, for as-prepared powders the values of Ca/(P + Se) molar ratios are between 1.67 and 1.80. It can be hypothesized that the incomplete incorporation of selenite ions is compensated by the substitution of PO_4_^3−^ with CO_3_^2^^−^, i.e., the formation of type-B carbonated apatites can be expected. As will be shown later, a significant amount of an amorphous phase (ACP) was observed in all as-prepared powders. The ACP can have a Ca/P molar ratio in the range 1.2–2.2, depending on the synthesis conditions and used precursors [4]. As shown in Table 3 the powder CaP_Se1 has the highest content of ACP phase. The higher Ca/P ratio of CaP_Se1 sample, compared to other powders, might be a result of the higher ACP content.

### 3.2. XRD Patterns of as-Prepared Powders and Rietveld Refinements

**Figure 1:**
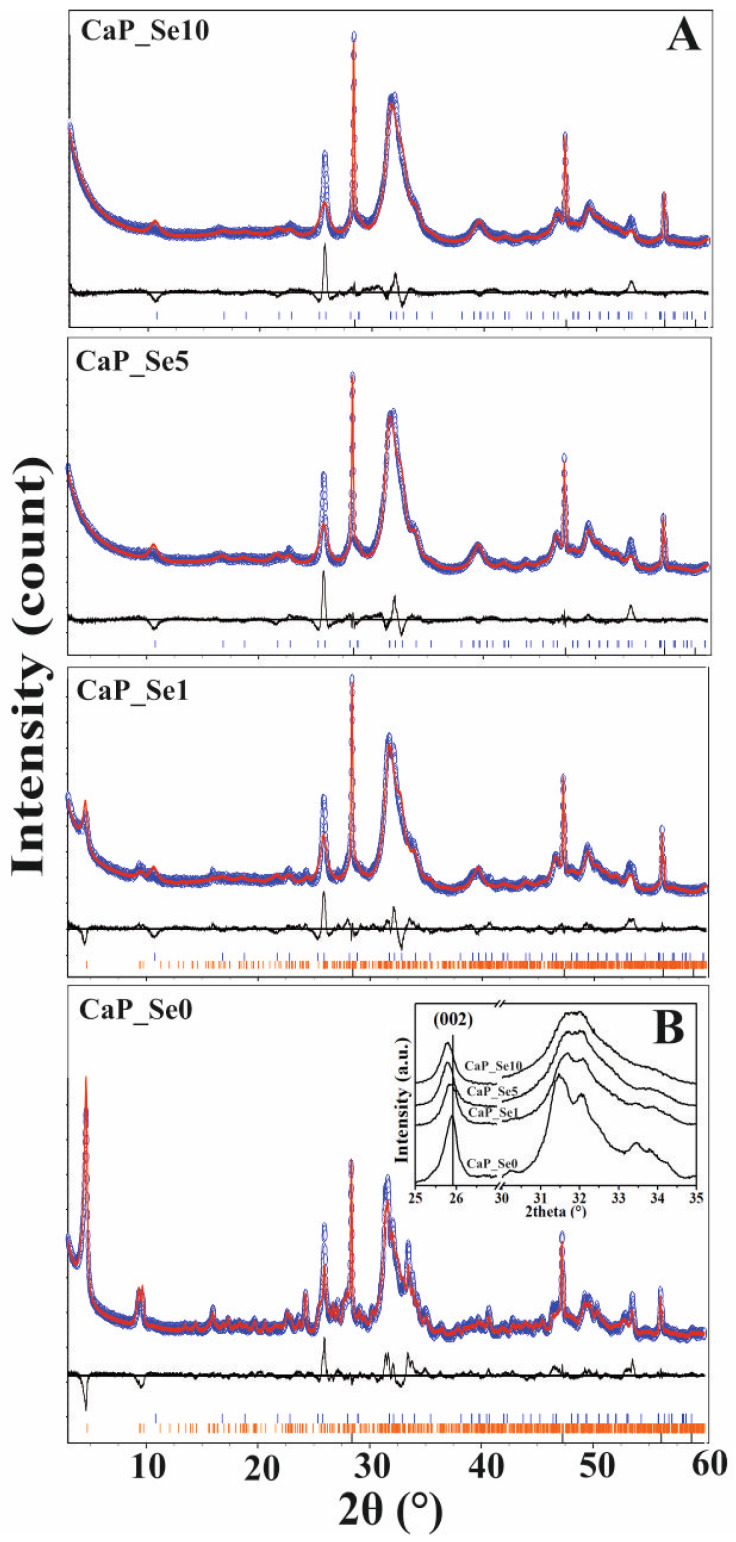
Rietveld analysis of powder X-ray diffraction data (**A**) and X-ray diffraction analysis patterns (**B**) for as-prepared non-substituted (CaP_Se0) and Se-substituted (CaP_Se1, CaP_Se5, CaP_Se10) CaP powders. Observed (blue empty circles) and calculated (red solid lines) intensities. The difference between the observed and calculated intensities is plotted below the profile (R_wp_ < 10%; R_exp_ < 3%). Bragg positions of hydroxyapatite, octacalcium phosphate, and silicon (standard) are marked below each pattern.

The XRD patterns of as-prepared non-substituted (CaP_Se0) and Se-substituted (CaP_Se1, CaP_Se5 and CaP_Se10) CaP powders are shown in Figure 1A,B. Samples CaP_Se0 and CaP_Se1 were identified as biphasic mixtures, with a good match to the line patterns for crystalline HAp (JCPDS No.09-0432) and OCP (JCPDS No. 27-1402), while CaP_Se5 and CaP_Se10 show characteristic diffraction peaks for HAp. No additional peaks characteristic for selenium compounds were observed. With the increase of the Se/P molar ratio, the characteristic diffraction peak intensity and the crystallinity decrease (Figure 1B), which indicates that incorporation of SeO_3_^2^^−^ leads to lattice distortion of obtained CaPs. Compared to the CaP_Se0, the diffraction peaks of the (002) crystal plane of the Se-substituted samples shifted to the left (Figure 1B). The peak shift of the (002) characteristic peak is related to the change of the HAp and/or OCP lattice constant *c*, which means that the incorporation of the SeO_3_^2^^−^ ions has a marked impact on the growth of the HAp crystal, particularly in the *c*-axis direction as previously described by Wei et al. [7].

Rietveld refinement analysis (Figure 1A) was performed on XRD patterns of all prepared samples to determine unit-cell parameters, crystallite size and weight percent proportions of detected phases. Results of quantitative analysis of samples are given in Table 3.

The Rietveld refinements indicate a significant amount of an ACP phase in all as-prepared powders (Table 3). The CaP_Se0 and CaP_Se1 powders in addition to HAp and ACP phase contain OCP phase as well. The ACP phase is a hydrated, thermodynamically unstable, transient phase that commonly precipitates during the formation of more stable CaPs in aqueous media. It is considered to be a precursor phase in OCP formation. The OCP is often found as an intermediate phase in the formation of thermodynamically more stable HAp. The final pH values (Table 3.) of all obtained suspensions were higher than 7. According to the literature [19], the calcium deficient hydroxyapatite (Ca/P = 1.5–1.67) can be obtained in the pH range 6.5 to 9.5, OCP (Ca/P = 1.33) in the pH range 5.5 to 7.0 and ACP (Ca/P = 1.0–2.2) in the pH range 5.0 to 12.0. The presence of OCP phase within CaP_Se0 and CaP_Se1 systems, even the pH value was higher than 7.0, suggest that foreign ions influence the phase transformation of calcium phosphates from amorphous to crystalline phases.

The lattice parameters, density, and average crystallite size of the HAp and OCP phase in as-prepared powders, determined by Rietveld refinement of the XRD data, are shown in Table 4.

**Figure 2:**
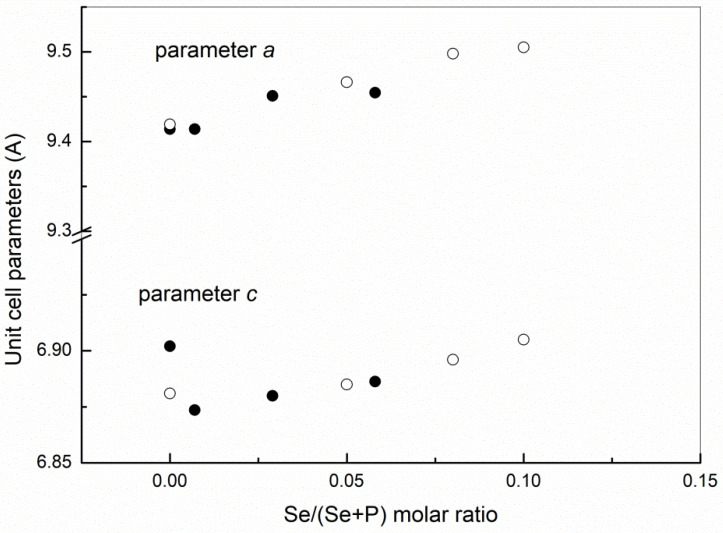
Unit cell parameters of HAp phase as a function of Se/(P + Se) molar ratio in as-prepared powders (black circles) compared to the literature data [20] (empty circles).

The variation of unit cell parameters of HAp phase as a function of Se/(P + Se) molar ratio in as prepared powders is shown in Figure 2.

Lattice parameter *a* (=*b*) increases with increasing Se-substitution, which can be explained by the larger bond length of Se‒O (0.164 nm) compared to P‒O bond length (0.155 nm). The unit cell parameter *c* of substituted HAp phase in all prepared powders is lower than *c* of non-substituted HAp but exhibited an increase with the Se substitution level. The lower value of the lattice parameter *c* in substituted HAp, compared to non-substituted HAps, may indicate the release of hydroxyl groups and formation of vacancies along the *c* axis due to charge compensation since SeO_3_^2^^−^ (double charged) substitute PO_4_^3−^ ion (triple charged), that consequently leads to Ca^2+^ and OH^−^ ion release [21,22]:

Ca_10_(PO_4_)_6_(OH)_2_ + *x*SeO_3_^2−^ → Ca_10-*x*_(PO_4_)_6−*x*_(SeO_3_)*_x_*(OH)_2−*x*_ + *x*PO_4_^3−^ + *x*Ca^2+^ + *x*OH^−^ (1)

The difference between the *c* parameters of non-substituted and Se-substituted HAps is smaller for higher Se-substitution levels, where the higher content of Na^+^ ions was detected, indicating additional charge defect compensation by Na-substitution for Ca according to the formula Ca_10-*x*_Na*_x_*(PO_4_)_6-*x*_(SeO_3_)*_x_*(OH)_2_. The lattice parameters of substituted samples compare fairly well with the literature data reported by Barbanente et al. [20], showing an almost linear rise of cell parameters with Se substitution up to a Se/(P + Se) molar ratio of 0.1 (Figure 2). Wei et al. [10] reported that *a* and *b* lattice parameters increase along with the Se-substitution level, while lattice parameter *c* decreases, while Sun et al. [21] reported a non-continuous change of *a* and *b* and decrease of *c* lattice parameters. Kolmas et al. [22] observed an increase of *a* and *b* and non-continuous change of *c* lattice parameters along with the Se-substitution level. The difference in the unit cell paramter *c* between the non-substituted (CaP_Se0) powder prepared in this work and those reported in the literature can be attributed to the biogenic precursor (cuttlefish bone) used in this work. The cuttlefish bone in its aragonite structure contains a significant amount of trace elements (e.g., Sr^2+^, Mg^2+^, Na^+^) that can influence the unit cell parameters of prepared CaPs.

As seen from Table 4, in spite of lower values of parameter *c* in substituted HAp, the unit cell volume, V, of the HAp phase in the prepared samples increases and the density consequently decreases with the Se-substitution level. Selenite ion (trigonal pyramidal) has greater volume compared to phosphate ion (tetrahedral) and consequently, the substitution may result in the dilatant unit cell and oxygen voids may be generated [10]. Very small difference in the average crystallite size L (ranging between 5 and 7 nm) was obtained for the HAp phase in the prepared non-substituted and Se-substituted samples.

The unit cell parameters, unit cell volume, and the average crystallite size of OCP phase in the powder CaP_Se1 differ from the values obtained for non-substituted CaP_Se0 powder, indicating the incorporation of selenite ions in the OCP phase, too. Since the powders CaP_Se5 and CaP_Se10 do not contain an OCP phase, the proper correlation between the mentioned parameters and selenite substitution level cannot be established. It should be noted that the Rietveld refinement is not sensitive enough to determine selenite concentration in each (HAp and OCP) phase.

### 3.3. XRD Patterns of Heat Treated Powders and Rietveld Refinements

**Figure 3:**
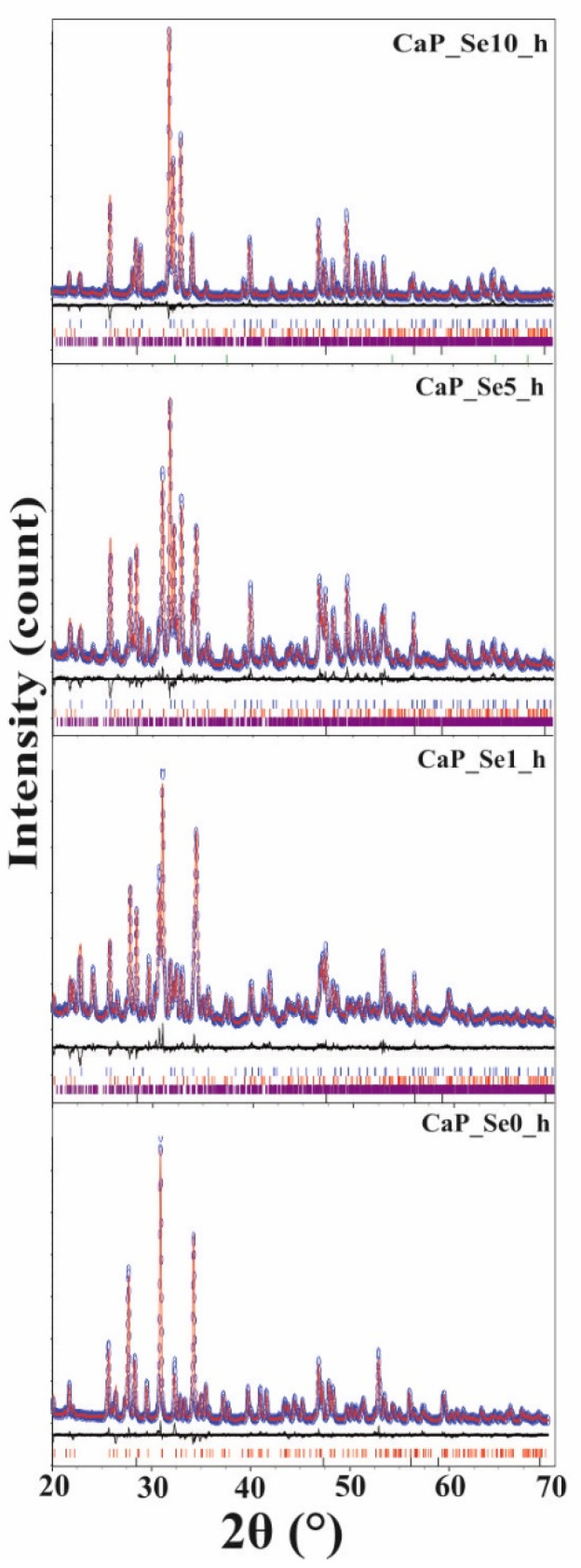
Rietveld analysis pattern of powder diffraction data for heat treated CaP powders. Observed (blue empty circles) and calculated (red solid lines) intensities. The difference between the experimental and calculated intensities is plotted below the profile (R_wp_ < 11%; R_exp_ < 3%). Bragg positions of hydroxyapatite, β-tricalcium phosphate, α-tricalcium phosphate, and silicon (standard) are marked below each pattern.

XRD patterns of heat treated powders at 1200 °C are presented in Figure 3.

Compared to the XRD patterns of as-prepared samples, XRD patterns sharpen after heat treatment, indicating an increase of crystallinity. The XRD data of the sample CaP_Se0 gave a good match to the line pattern of *β*-TCP (JCPDS No. 09-0169). Samples CaP_Se1, CaP_Se5, and CaP_Se10 were identified as triphasic mixtures with a good match to the patterns of HAp (JCPDS No.09-0432), *β*-TCP, and *α*-TCP (JCPDS No. 09-0348). Results of the quantitative phase analysis of the heat-treated samples determined by Rietveld refinement of the XRD data are given in Table 5.

With the increase of selenite ion content, an increase in the HAp content and a decreasing trend in *β*-TCP and *α*-TCP content have been observed. Similar findings that the incorporation of selenite ions restricts the phase transformation from HAp to *β*-TCP are reported in the literature [7,10,23]. As observed by Wei et al. [10] the samples with Se/P ≤ 0.05 did not transform to the TCP phase after heat treatment at 1100 °C.

As seen from Table 5 in the heat-treated sample CaP_Se10 CaO phase was detected, as well. Wei et al. [10] observed CaO as secondary phase after high-temperature sintering of the sample prepared with a Se/P molar ratio of 0.08. They speculate that the CaO is formed by transformation of CaSeO_3_ that eventually precipitates in a very small amount and cannot be detected by XRD.

### 3.4. FTIR Analysis

**Figure 4:**
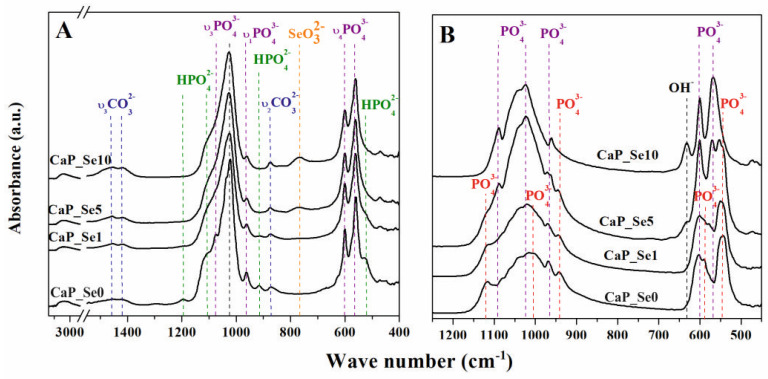
FTIR spectra of as-prepared (**A**) and heat-treated (**B**) non-substituted (CaP_Se0) and Se-substituted (CaP_Se1, CaP_Se5, CaP_Se10) CaP powders.

The FTIR spectra of as-prepared and heat treated CaP powders are shown in Figure 4A,B, respectively.

FTIR spectra of as-prepared samples show broad bands in the range 3000‒3700 cm^−1^, attributed to adsorbed water. The strong bands in the range 900‒1200 cm^−1^ correspond to characteristic stretching vibration modes of the phosphate group. The bands at 1022 and 1074 cm^−1^ (ν_3_) are attributed to the asymmetric stretching vibration of P–O, at 964 cm^−1^ (ν_1_) is associated with the symmetric stretching vibration of P–O, and at 565 and 599 (ν_4_) cm^−1^ are attributed to asymmetric bending vibrations of O–P–O, all of which can be assigned to the HAp phase [24]. Absorption bands at 525, 912, 1112, and 1193 cm^−1^, which are attributed to the characteristic absorption bands of a HPO_4_^2−^ group, can be assigned to the OCP and ACP phases [25,26,27,28,29]. The intensities of HPO_4_^2−^ bands decrease along with the increase of Se-substitution level, since the content of OCP and ACP phase decrease, as well. The bands at 875, 1419, and 1463 cm^−1^ are characteristic vibration modes for carbonate groups substituted for phosphate group (B-type substitution) [30,31]. CO_3_^2−^ substitution was expected since CaCO_3_ was used as the source of Ca-ions. Additionally, it could be the result of CO_2_ dissolution in the reaction medium during the open-air synthesis. The band at 765 cm^−1^ is attributed to O‒Se‒O bending vibration (ν_3_) and it becomes stronger as the Se-substitution level increases [22]. The characteristic vibration modes for Se‒O bands are in the range of 900‒800 cm^−1^ [23]. However, they overlap with vibration modes of carbonate groups. Obtained results indicate that prepared samples are co-substituted with SeO_3_^2−^ and CO_3_^2−^ in the position of PO_4_^3−^ ions. CO_3_^2−^ ions are considered to be more easily incorporated into the HAp lattice due to having a smaller volume than SeO_3_^2−^ ions [10,32].

In the FTIR spectra of the heat-treated samples (Figure 4B) the band at 631 cm^−1^ is assigned to the hydroxyl group of HAp. Its intensity increases as the Se/P ratio increases, in agreement with the quantitative analysis of the heat-treated CaP powders performed by Rietveld refinement of the XRD data that shows stabilization of the HAp phase during the heat treatment process. Characteristic P‒O bands of HAp appear at 1090, 1023, 965, 600, and 568 cm^−1^. A broad band from 950 to 1200 cm^−1^ corresponds to the TCP phases. The bands at 1120 and 1004 cm^−1,^ attributed to stretching vibration of P‒O, at 940 cm^−1^, associated with banding modes of P‒O, at 1040, 1062, and 1082 cm^−1^ (*ν*_3_), attributed to anti-symmetric P‒O stretching, and at 542, 588, and 600 cm^−1^ (*ν*_3_), attributable to anti-symmetric P‒O bending can be assigned to *β*-TCP phase. The bands at 940 and 965 cm^−1^ arise due to the factor group splitting of the ν_1_ fundamental vibration mode corresponding to the symmetric P‒O stretching vibration of the phosphate ion (958 cm^−1^). The characteristic bands for *β*-TCP are similar to bands of *α*-TCP, however, the latter are broader [33]. This effect can be seen in the FTIR spectrum for CaP_Se1 compared to CaP_Se0 powder. Characteristic band at 765 cm^−1^, attributed to O‒Se‒O bending vibration, disappears as a result of decomposition of selenite with the release of SeO_2_ in gas form as previously reported by Wei et al. [10].

### 3.5. SEM Analysis

**Figure 5:**
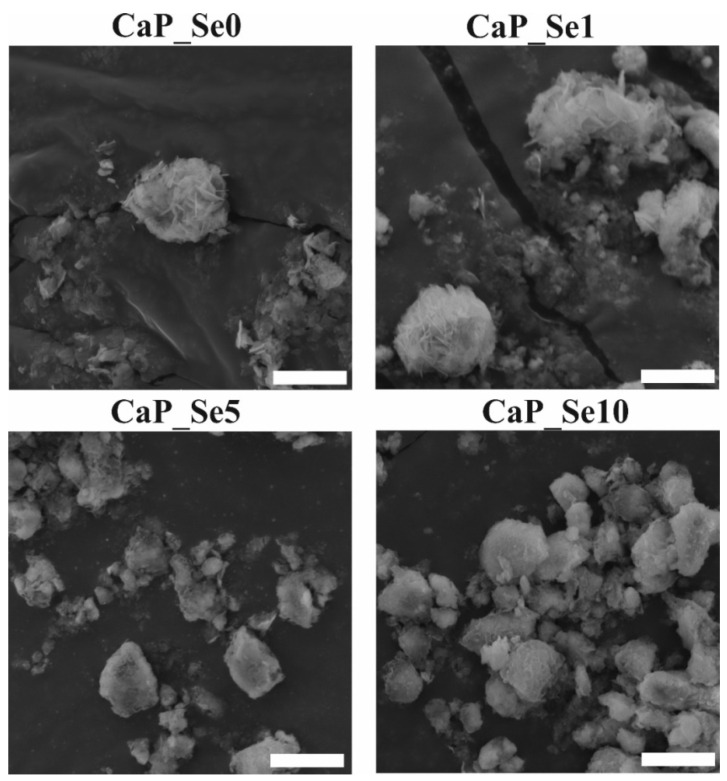
SEM micrographs of as-prepared CaP powders. Scale bars: 5 μm.

SEM micrographs of as-prepared powders are given in Figure 5, showing both spherical particles and irregularly shaped agglomerates. The surface of particles in CaP_Se0 and CaP_Se1 powders exhibited plate like morphology, typical for OCP and frequently observed for synthetic and natural apatites. It seems that the higher Se-substitution level in CaP_Se5 and CaP_Se10 samples resulted in a reduced particle size and the plate like crystals on the particle surface are less evident.

From the obtained SEM images, it is difficult to estimate the average size of the particles since they are not well dispersed. It seems that a fraction of particles settled at the bottom, and it is difficult to distinguish between particle boundaries. The size of spherical agglomerates in CaP_Se0 and CaP_Se1 powders is around 5 μm, while the agglomerates in CaP_Se5 and CaP_Se10 powders are smaller than 5 μm.

### 3.6. Ion Release and In Vitro Bioactivity

**Figure 6:**
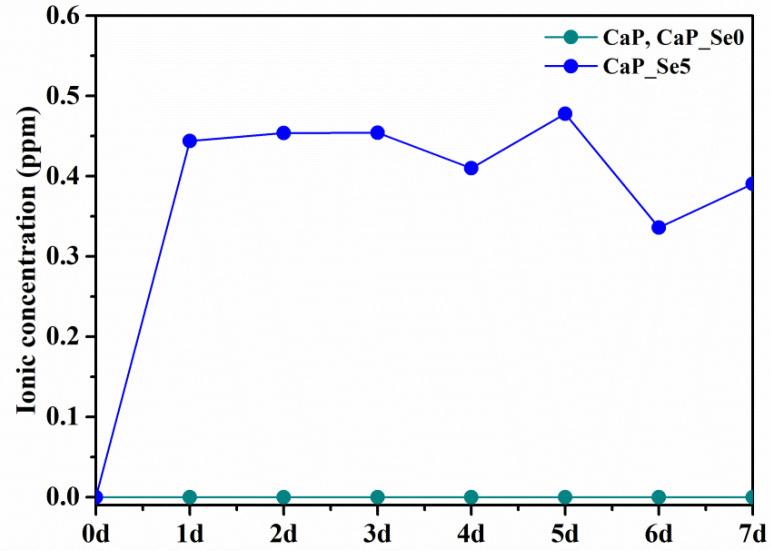
Released SeO_3_^2−^ ion concentration during 7 days of incubation at 37 °C.

The dissolution of the as-prepared powders and their ability to form apatite like phases in simulated body conditions were followed by ICP-MS and XRD analysis. Release curve of selenite ions for CaP_Se5 powder shown in Figure 6 indicate an initial burst release (day 1) followed by a stage of slow and mostly steady release. The pH value of the SBF solution varied slightly during incubation time from an initial 7.40 to a maximum of 7.66.

The concentration of released Se ion is the same for CaP and CaP_Se0 powder (selenite-free samples, 0 ppm, green line in Figure 6).

Results of quantitative XRD analysis of the powders CaP_Se0 and CaP_Se5 after 7 days of immersion in SBF, performed by Rietveld refinement, are given in Table 6.

As seen, during soaking the partial transformation of OCP and ACP phases into the HAp phase occurred. When apatite samples are immersed in SBF, both dissolution and the precipitation of apatite occurs simultaneously. SBF solution contains calcium, phosphate and carbonate ions, the concentrations of which are close to the solubility limit of carbonated HAp. The dissolution and liberation of ions can result in localized supersaturation that favors precipitation of carbonated HAp. From XRD patterns it was not possible to distinguish between the original powder phases and the newly formed apatite phase.

### 3.7. Selective Anticancer Activity of Selenium Substituted Calcium Phosphates

**Figure 7:**
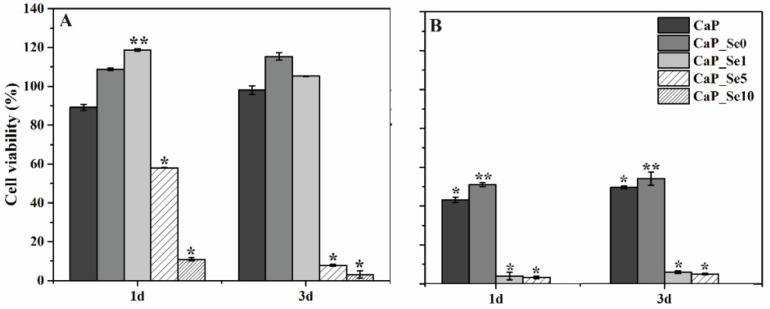
The effect of supernatant of calcium phosphate powders on cell viability (%) of HEK 293 (**A**) and U2OS (**B**) cells, respectively. The significant difference between two groups: ** (*p* < 0.05), * (*p* < 0.01).

The main goal of preparing selenium substituted CaPs is obtaining a biomaterial that will effectively inhibit the development of bone cancers while showing no cytotoxic activity on healthy cells. The cell viability results are given in Figure 7.

Figure 7A shows that in comparison with the control group (untreated cells), in which cell viability was estimated to be 100%, extracts of CaP_Se0 and CaP_Se1 powders exert a proliferative effect on the HEK 293 cells. The higher cell viability for CaP_Se0 compared to CaP (non-substituted CaP powder obtained from synthetic CaCO_3_) indicates a beneficial effect of foreign ions present in the biogenic Ca-ion precursor on cell proliferation. The treatment with extracts of CaP_Se5 for 1 and 3 days reduced HEK 293 cell viability by ~40 and ~90%, respectively, while the CaP_Se10 powder reduced HEK 293 cell viability by ~90% or more, at both time points. The anticancer activity was examined using U2OS cell line. As Figure 7B shows, the cell proliferation was significantly suppressed in the presence of the extracts of all investigated powders. The extracts of CaP and CaP_Se0 powders caused a significant reduction in the U2OS cell viability (50% and 54%, respectively) while the extracts of the CaP_Se1 and CaP_Se5 powders reduced U2OS cell viability by more than 95% compared to untreated cells.

Uskoković et al. [9] also reported that the viability of K7M2 osteosarcoma cells decreased in direct proportion to the amount of selenite in HAp but no reduction was observed in the viability of primary fibroblasts treated with HAp incorporating different amounts of selenite ions, suggesting their potentially selective anticancer activity. Our results suggest that only the CaP_Se1 powder, with low concentration of Se, show selective toxicity to cancer cells (U2OS), without harming non-tumorigenic cells (HEK 293) cells. Results are consistent with the study of Barbanente et al. [20], who evaluated the cytotoxic activity of Se-doped HAp nano particles using prostate and breast cancer cells as well as healthy human bone marrow stem cells. They found that nanoparticles with a high concentration of Se showed a strong anticancer effect but also caused a significant increase in toxicity towards normal cells.

Additional in vitro osteogenic cell culture experiments and in vivo studies are needed to confirm the suitability of investigated materials for potential application in bone tumor therapy. Furthermore, in our future research, highly porous scaffolds based on a biopolymer and selenite substituted CaPs will be prepared and characterized.

## 4. Conclusions

In the present study, Se-substituted calcium phosphates have been prepared via an aqueous precipitation reaction at mild conditions. As a result of using biogenic source (cuttlefish bone) as a precursor of Ca^2+^ ions along with the selenite substitution, the presence of Sr^2+^, Na^+^, and Mg^2+^ was detected by ICP-MS analysis. The selenium ion concentration affects the phase composition of obtained powders, consisting of calcium deficient bone-like HAp, OCP, and ACP. The presence of selenium ions in as-prepared powders stabilizes the HAp phase during heat treatment at 1200 °C. The CaP_Se1powder with the Se/(P + Se) molar ratio of 0.007 showed selective toxicity to cancer cells. The treatment with extracts of CaP_Se5 powder (Se/(P + Se) molar ratio of 0.029) for 1 and 3 days reduced HEK 293 cell viability for ~40 and ~90%, respectively. The CaP_Se10 powder with Se/(P + Se) molar ratio of 0.058 reduced HEK 293 cell viability for ~90% at both time points. The extracts of CaP and CaP_Se0 (selenium-free) powders caused a significant reduction in the U2OS cell viability (50% and 54%, respectively) while the extracts of the CaP_Se1 and CaP_Se5 powders reduced U2OS cell viability by more than 95%, compared to untreated cells.

## Figures and Tables

**Table 1:** Quantities of reactants and nominal composition of Se-substituted calcium phosphates.

	CaCO_3_ (mmol)	UPH (mmol)	Na_2_SeO_3_·5H_2_O (mmol)	Nominal Se/(P + Se)
CaP_Se0	4.977	2.985	0.000	0.00
CaP_Se1	4.977	2.955	0.030	0.01
CaP_Se5	4.977	2.834	0.149	0.05
CaP_Se10	4.977	2.687	0.299	0.10

**Table 2:** Results of ICP-MS analysis.

Sample	Minor Substituents (mol%)			Se/(P + Se) Molar Ratio	Ca/(P + Se) Molar Ratio
	Sr	Na	Mg		
CaP_Se0	0.46	1.45	0.44	0.000	1.67
CaP_Se1	0.43	1.97	0.44	0.007	1.80
CaP_Se5	0.48	3.23	0.46	0.029	1.68
CaP_Se10	0.50	3.49	0.44	0.058	1.68

**Table 3:** Quantitative analysis of CaP phases in as-prepared powders performed by Rietveld refinement of the XRD data and the final pH values of CaPs suspensions measured at room temperature (T = 22.0 ± 0.5).

		wt%		pH
Sample	HAp	OCP	ACP	
CaP_Se0	27.8	43.9	28.3	7.60
CaP_Se1	61.2	8.7	30.1	7.33
CaP_Se5	76.7	-	23.3	8.00
CaP_Se10	77.9	-	22.1	8.63

**Table 4:** Unit-cell parameters of HAP and OCP phase obtained by Rietveld refinement of the XRD data.

	Sample Codes	Structural Parameters					
		V (Å^3^)	*a* (Å)	*b* (Å)	*c* (Å)	φ (g/cm^3^)	L (nm)
**HAp**	CaP_Se0	529.69(1)	9.41369	9.41369	6.90197	4.233	5.15
	CaP_Se1	531.69(6)	9.41371	9.41371	6.87351	3.131	7.33
	CaP_Se5	532.56(5)	9.45098	9.45098	6.87991	3.126	6.65
	CaP_Se10	533.55(9)	9.45430	9.45430	6.88637	3.120	5.79
**OCP**	CaP_Se0	1220.96(1)	19.69511	9.53534	6.85167	2.780	33.85
	CaP_Se1	1226.91(8)	19.71504	9.50590	6.89569	2.660	18.19

**Table 5:** Results of quantitative analysis of the heat treated CaP powders performed by Rietveld refinement of the XRD data.

Sample	wt%				
	HAp	*β*-TCP	*α*-TCP	ACP	CaO
CaP_Se0	-	88.6	-	11.4	-
CaP_Se1	10.6	50.4	32.5	6.5	-
CaP_Se5	45.8	36.6	9.8	7.8	-
CaP_Se10	88.2	3.1	4.1	4.4	0.2

**Table 6:** Results of quantitative analysis of powders after soaking in SBF compared to as-prepared powders.

	As-Prepared Powders			After Soaking in SBF		
Sample	HAp	OCP	ACP	HAp	OCP	ACP
CaP_Se0	27.8	43.9	28.3	45.0	27.9	27.1
CaP_Se5	76.7	-	23.3	86.3	-	13.7

## Data Availability

Data sharing is not applicable to this article.
